# Supplementary material for: The parietal operculum preferentially encodes heat pain and not salience
Source: PLoS Biol. 2019 Aug 12;17(8):e3000205. doi: 10.1371/journal.pbio.3000205 (PMC6705876; doi:10.1371/journal.pbio.3000205)
Supplement: S1 Table — (DOCX) [file pbio.3000205.s001.docx]

| **Questionnaire** | **Construct** | **Mean±SD** | **Sample range** | **Possible range** |
| --- | --- | --- | --- | --- |
| **BDI-II** [1,2] | Depression | 2.0±2.2 | 0-8 | 0-63 |
| **PHQ15** [3] | Somatization | 3.9±2.3 | 0-9 | 0-30 |
| **FPQ** [4] |  |  |  |  |
| severe | Fear of pain | 28.9±8.3 | 11-42 | 10-50 |
| minor | Fear of pain | 14.0±4.5 | 10-27 | 10-50 |
| **PVAQ** [5] | Pain vigilance and awareness | 34.6±8.7 | 21-54 | 0-80 |
| **PSQ** [6] | Pain sensitivity | 45.1±14.8 | 17-73 | 0-140 |
| **PRSS** [7] |  |  |  |  |
| Catastrophizing | Pain catastrophizing | 8.6±5.4 | 2-21 | 0-45, higher more catastrophizing |
| Coping | Pain coping | 29.8±5.2 | 19-38 | 0-45, higher more active coping |
| **STAI** [8,9] |  |  |  |  |
| Trait | Trait anxiety | 32.0±6.9 | 21-49 | 20-80 |
| State | State anxiety (pre experiment) | 31.5±6.2 | 22-51 | 20-80 |
| **MDMQ** [10] |  |  |  |  |
| GoodBad A | Mood: Good vs bad (pre exp.) | 17.7±1.9 | 12-20 | 4-24, the higher the better |
| AwakeTired A | Mood: Awake vs tired (pre exp.) | 15.2±2.9 | 8-19 | 4-24, the higher the more awake |
| CalmNervous A | Mood: Calm vs nervous (post exp.) | 16.5±2.6 | 10-20 | 4-24, the higher the calmer |
| GoodBad B | Mood: Good vs bad (pre exp.) | 17.2±1.7 | 12-19 | 4-24, the higher the better |
| AwakeTired B | Mood: Awake vs tired (pre exp.) | 10.1±2.4 | 6-16 | 4-24, the higher the more awake |
| CalmNervous B | Mood: Calm vs nervous (post exp.) | 17.2±2.1 | 12-20 | 4-24, the higher the calmer |

References.

1. Beck AT, Steer RA, Brown GK. Manual for the Beck Depression Inventory-II. San Antonio, TX: Psychological Corporation Press; 1996.

2. Hautzinger M, Keller F, Kühner C. BDI-II. Beck-Depressions-Inventar. Revision. 2nd ed. Frankfurt: Pearson Assessment; 2009.

3. Kroenke K, Spitzer RL, Williams JBW. The PHQ-15: validity of a new measure for evaluating the severity of somatic symptoms. Psychosom Med. 2002;64(2):258–66.

4. McNeil DW, Rainwater AJ. Development of the Fear of Pain Questionnaire-III. J Behav Med. 1998;21(4):389–410.

5. McCracken LM. “Attention” to pain in persons with chronic pain: A behavioral approach. Behav Ther. 1997;28(2):271–84. doi: 10.1016/S0005-7894(97)80047-0

6. Ruscheweyh R, Marziniak M, Stumpenhorst F, Reinholz J, Knecht S. Pain sensitivity can be assessed by self-rating: Development and validation of the Pain Sensitivity Questionnaire. Pain. 2009;146(1):65–74. doi: 10.1016/j.pain.2009.06.020

7. Flor H, Behle DJ, Birbaumer N. Assessment of pain-related cognitions in chronic pain patients. Behav Res Ther. 1993;31(1):63–73.

8. Spielberger CD, Gorsuch RL, Lushene RE. Manual for the State-Trait Anxiety Inventory. Palo Alto, CA: Consulting Psychologists Press; 1970.

9. Laux L, Glanzmann P, Schaffner P, Spielberger CD. Das State-Trait-Angstinventar. Weinheim: Beltz; 1981.

10. Steyer R, Schwenkmezger P, Notz P, Eid M. Der Mehrdimensionale Befindlichkeitsfragebogen (MDBF). Handanweisung. Göttingen: Hogrefe; 1997.
