# Supplementary material for: The parietal operculum preferentially encodes heat pain and not salience
Source: PLoS Biol. 2019 Aug 12;17(8):e3000205. doi: 10.1371/journal.pbio.3000205 (PMC6705876; doi:10.1371/journal.pbio.3000205)
Supplement: S2 Table — Cohort 2 received higher intensities; see Materials and methods for rationale. (DOCX) [file pbio.3000205.s002.docx]

|  | **Cohort 1 (n=15)** |  |  | **Cohort 2 (n=11)** |  |  |
| --- | --- | --- | --- | --- | --- | --- |
| **Intensity** | **Target VAS** | **dBA mean±SD** | **Range** | **Target VAS** | **dBA mean±SD** | **Range** |
| 1 | 25 | 73.3±5.4 | 61.0-82.9 | 48 | 85.0±9.0 | 65.7-99.3 |
| 2 | 35 | 77.0±4.9 | 67.7-85.9 | 59 | 91.3±8.3 | 71.1-101.5 |
| 3 | 45 | 80.5±5.0 | 73.6-89.6 | 70 | 95.1±7.6 | 76.3-102.5 |
| 4 | 55 | 83.8±5.4 | 76.7-96.1 | 82 | 97.6±6.1 | 81.7-103.0 |
| 5 | 65 | 86.6±5.4 | 78.9-99.3 | 93 | 99.3±4.8 | 86.3-103.0 |
| 6 | 75 | 89.1±5.2 | 81.0-100.5 | 105 | 100.6±3.5 | 91.0-103.0 |
