# Supplementary material for: The parietal operculum preferentially encodes heat pain and not salience
Source: PLoS Biol. 2019 Aug 12;17(8):e3000205. doi: 10.1371/journal.pbio.3000205 (PMC6705876; doi:10.1371/journal.pbio.3000205)
Supplement: S3 Table — SCR, skin conductance response. (DOCX) [file pbio.3000205.s003.docx]

| **Measure** | **Mean±SD of n=26 included subjects^1^** | **Mean±SD of n=32 excluded subjects** | **Mean differences^2^** | **Variance differences^3^** |
| --- | --- | --- | --- | --- |
| Heat pain threshold | 43.5±1.1 | 43.3±1.1 | t(56)=0.496, p=0.622 | F(31,25)=1.000, p=0.989 |
| Heat intensity 1 | 41.9±1.4 | 41.7±1.4 | t(56)=0.499, p=0.620 | F(31,25)=1.094, p=0.825 |
| Heat intensity 2 | 42.5±1.3 | 42.3±1.2 | t(56)=0.508, p=0.613 | F(31,25)=1.066, p=0.879 |
| Heat intensity 3 | 43.1±1.1 | 43.0±1.1 | t(56)=0.505, p=0.615 | F(31,25)=1.024, p=0.961 |
| Heat intensity 4 | 43.8±1.0 | 43.7±1.0 | t(56)=0.480, p=0.633 | F(31,25)=0.975, p=0.936 |
| Heat intensity 5 | 44.4±1.0 | 44.3±1.0 | t(56)=0.424, p=0.673 | F(31,25)=0.930, p=0.840 |
| Heat intensity 6 | 45.1±1.0 | 45.0±1.1 | t(56)=0.343, p=0.733 | F(31,25)=0.905, p=0.785 |
| Sound unpleasantness threshold | 82.9±7.3 | 83.9±7.0 | t(56)=-0.537, p=0.593 | F(31,25)=1.079, p=0.853 |
| Sound intensity 1 | 76.8±10.4 | 78.3±9.2 | t(56)=-0.552, p=0.583 | F(31,25)=1.283, p=0.528 |
| Sound intensity 2 | 81.9±9.9 | 83.1±9.6 | t(56)=-0.454, p=0.651 | F(31,25)=1.063, p=0.885 |
| Sound intensity 3 | 86.2±9.8 | 86.7±9.5 | t(56)=-0.206, p=0.838 | F(31,25)=1.055, p=0.901 |
| Sound intensity 4 | 89.6±9.1 | 89.6±8.9 | t(56)=-0.004, p=0.997 | F(31,25)=1.041, p=0.928 |
| Sound intensity 5 | 92.4±8.0 | 92.0±8.1 | t(56)=0.172, p=0.864 | F(31,25)=0.973, p=0.932 |
| Sound intensity 6 | 94.7±6.9 | 94.0±7.3 | t(56)=0.369, p=0.714 | F(31,25)=0.899, p=0.771 |

^1^Included subjects pooled over both cohorts; ^2^Two-sample t-test; ^3^Two-sample F-test
