# Supplementary figures and images for: The parietal operculum preferentially encodes heat pain and not salience
Source: PLoS Biol. 2019 Aug 12;17(8):e3000205. doi: 10.1371/journal.pbio.3000205 (PMC6705876; doi:10.1371/journal.pbio.3000205)

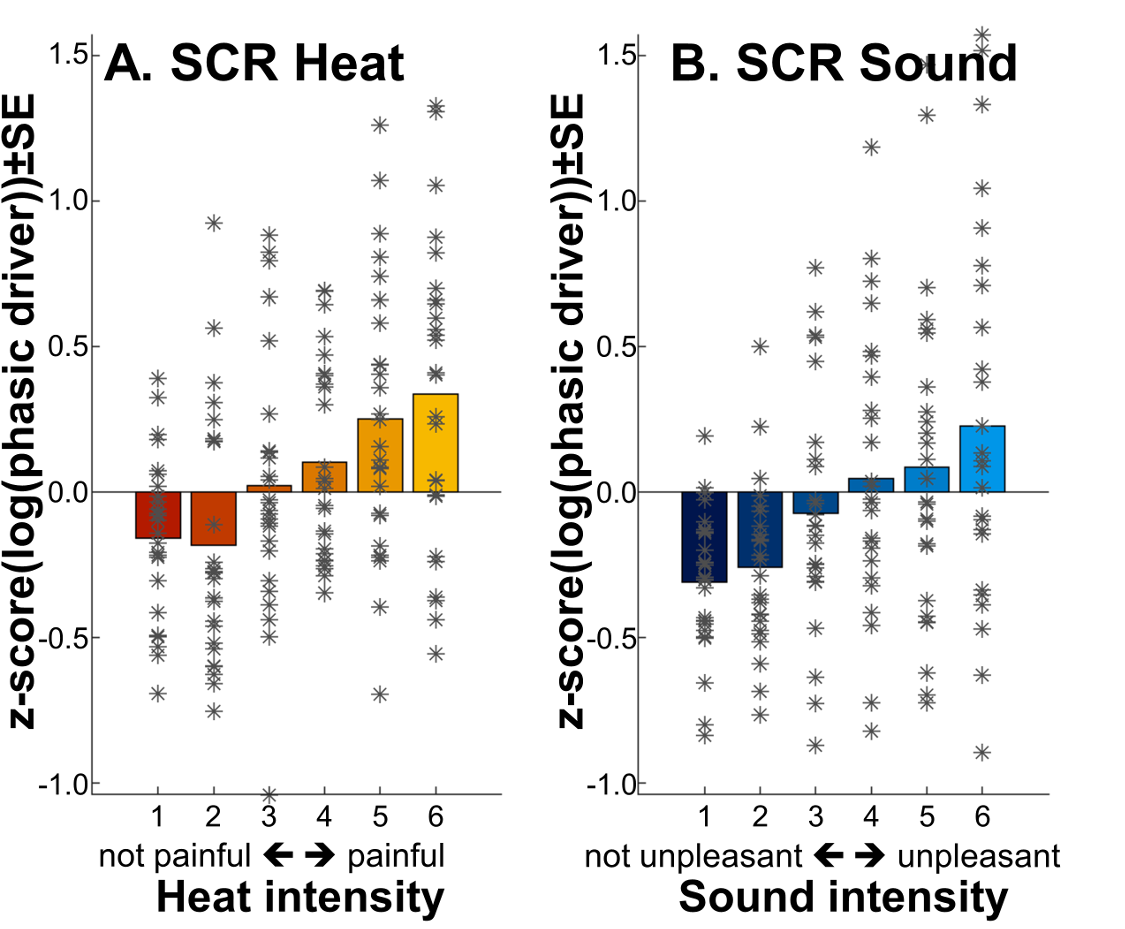

Supplement: S1 Fig — SCRs following heat (A) and sound (B) stimuli, with individual data points. The pain and unpleasantness thresholds were located between intensities 3 and 4, as per calibration. Data used to produce the figure can be found at https://www.doi.org/10.17605/OSF.IO/QXCNW within the component “Rating and SCR data.” SCR, skin conductance response. (TIF) [file pbio.3000205.s004.tif]

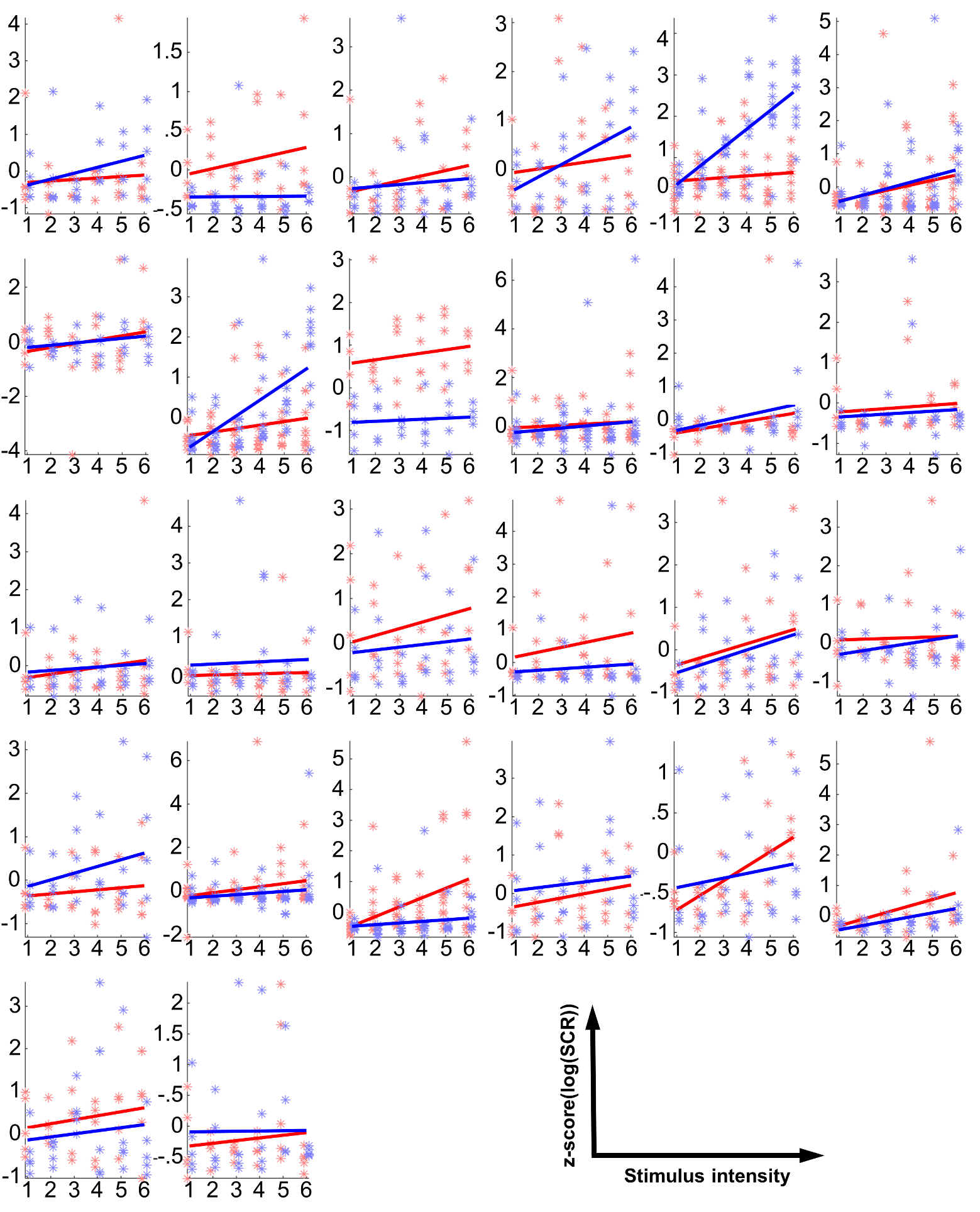

Supplement: S2 Fig — Asterisks indicate single responses, and lines are regression curves as per linear regression. The pain and unpleasantness thresholds were located between intensities 3 and 4, as per calibration. Data used to produce the figure can be found at https://www.doi.org/10.17605/OSF.IO/QXCNW within the component “Rating and SCR data.” SCR, skin conductance response. (TIF) [file pbio.3000205.s005.tif]

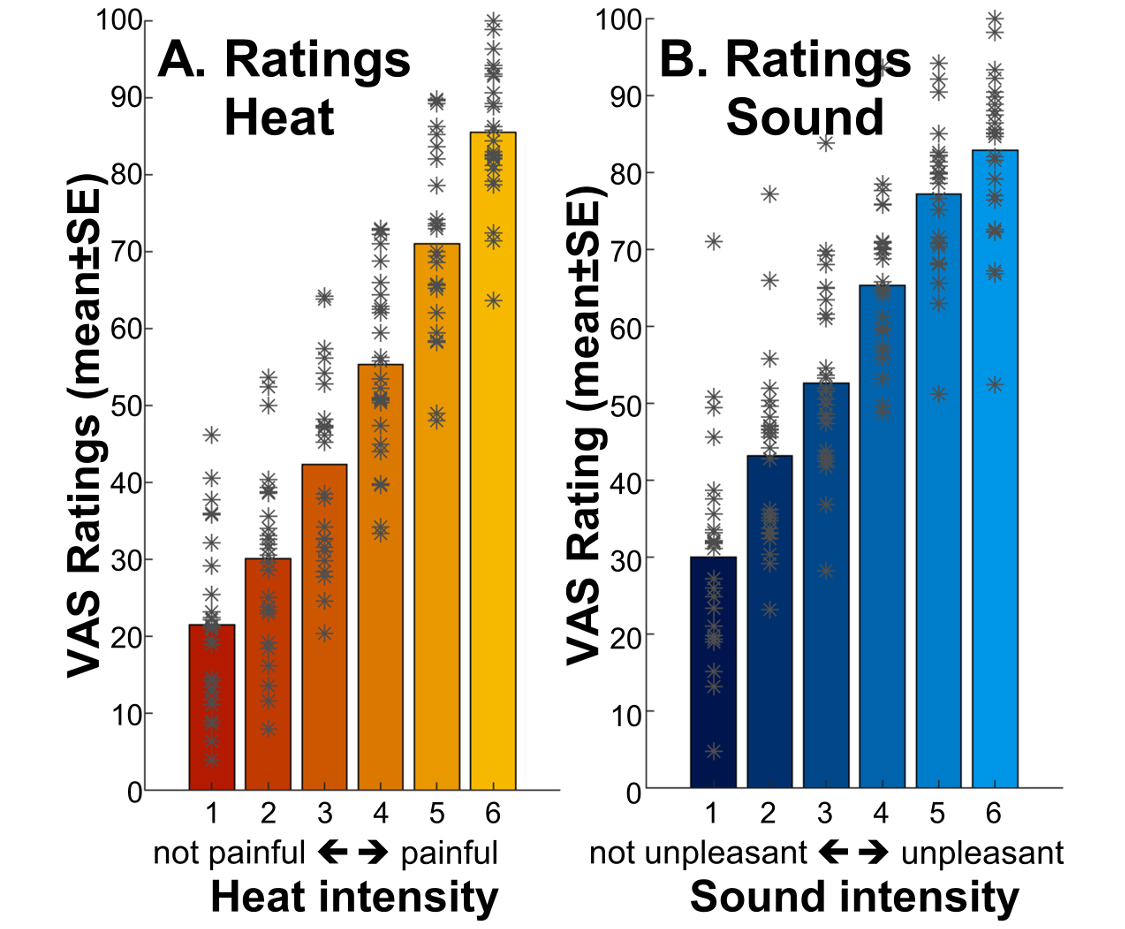

Supplement: S3 Fig — Behavioral ratings following heat (A) and sound (B) stimuli, with individual data points. The pain and unpleasantness thresholds were located between intensities 3 and 4, as per calibration. Data used to produce the figure can be found at https://www.doi.org/10.17605/OSF.IO/QXCNW within the component “Rating and SCR data.” (TIF) [file pbio.3000205.s006.tif]

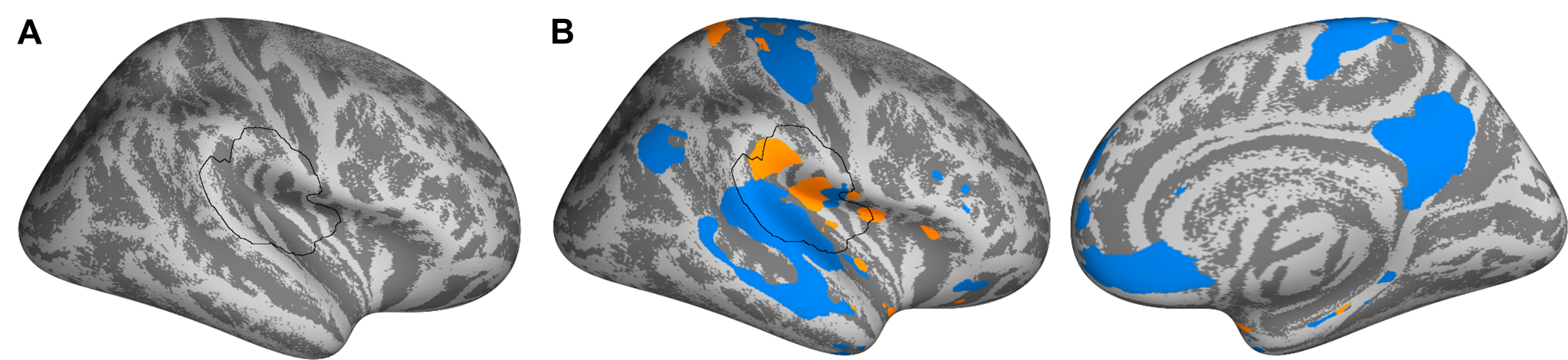

Supplement: S4 Fig — (A) Binary mask used for small-volume correction used for all analyses (unless otherwise noted), delineated by the black line. (B) Signed mask used for covering heat (orange) or sound (blue) contrasts. (TIF) [file pbio.3000205.s007.tif]

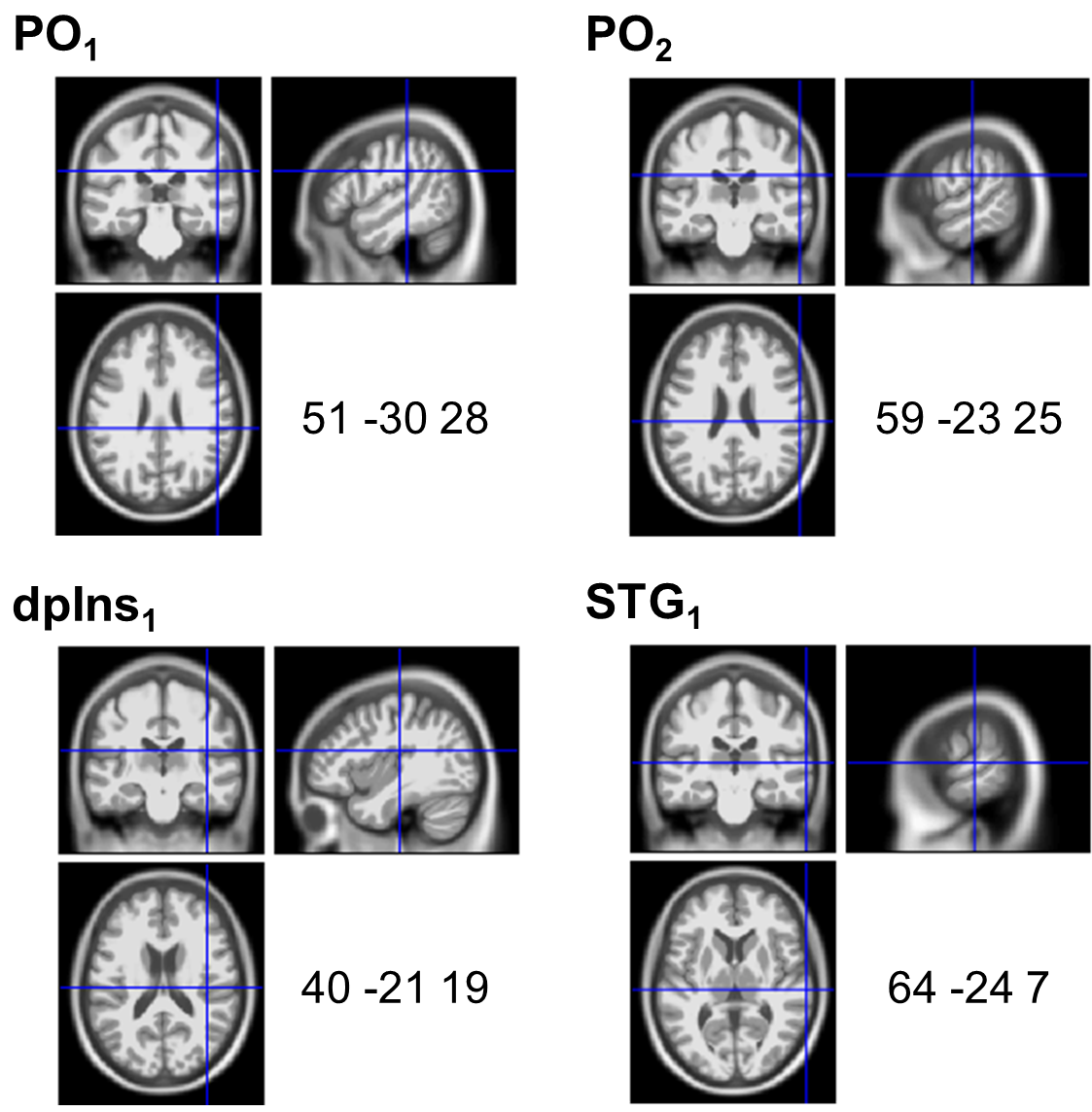

Supplement: S5 Fig — Activation was located in PO1, PO2, and dpIns1 for heat, and STG1 for sound. Also see Fig 4. dpIns1, dorsal posterior insula cluster 1; PO1, parietal operculum cluster 1; STG1, superior temporal gyrus cluster 1. (TIF) [file pbio.3000205.s008.tif]

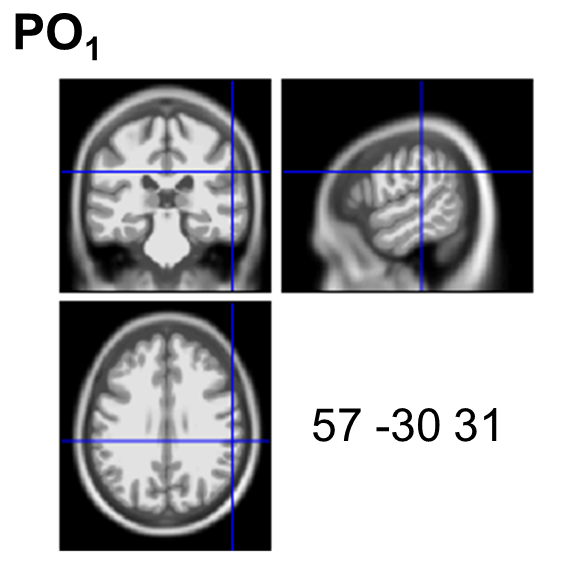

Supplement: S6 Fig — Also see Fig 5. PO1, parietal operculum cluster 1. (TIF) [file pbio.3000205.s009.tif]

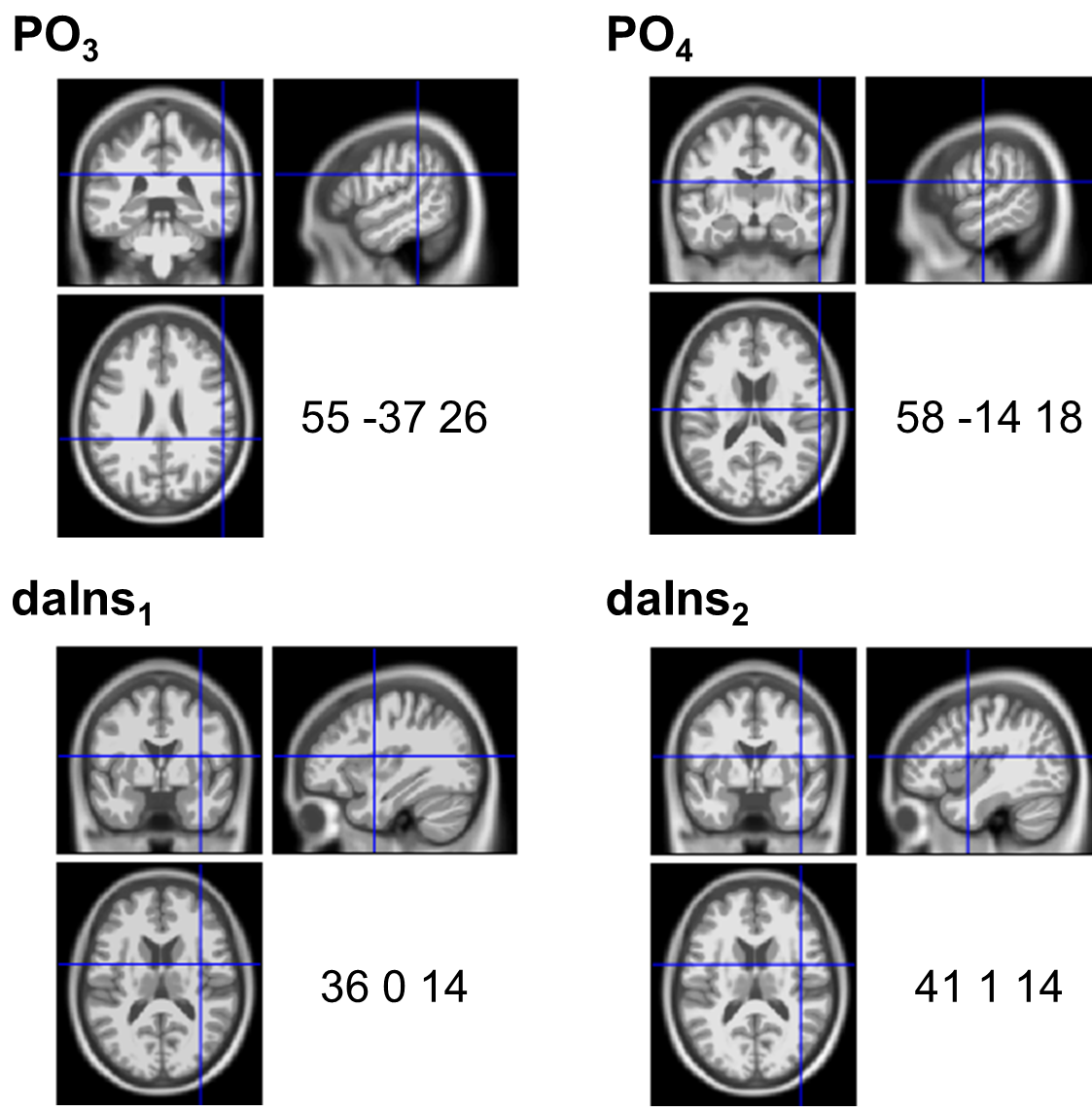

Supplement: S7 Fig — Activation was located in PO3, PO4, daIns1, and daIns2. Also see Fig 6. daIns1, dorsal anterior insula cluster 1; daIns2, dorsal anterior insula cluster 3; PO3, parietal operculum cluster 3; PO4, parietal operculum cluster 4. (TIF) [file pbio.3000205.s010.tif]

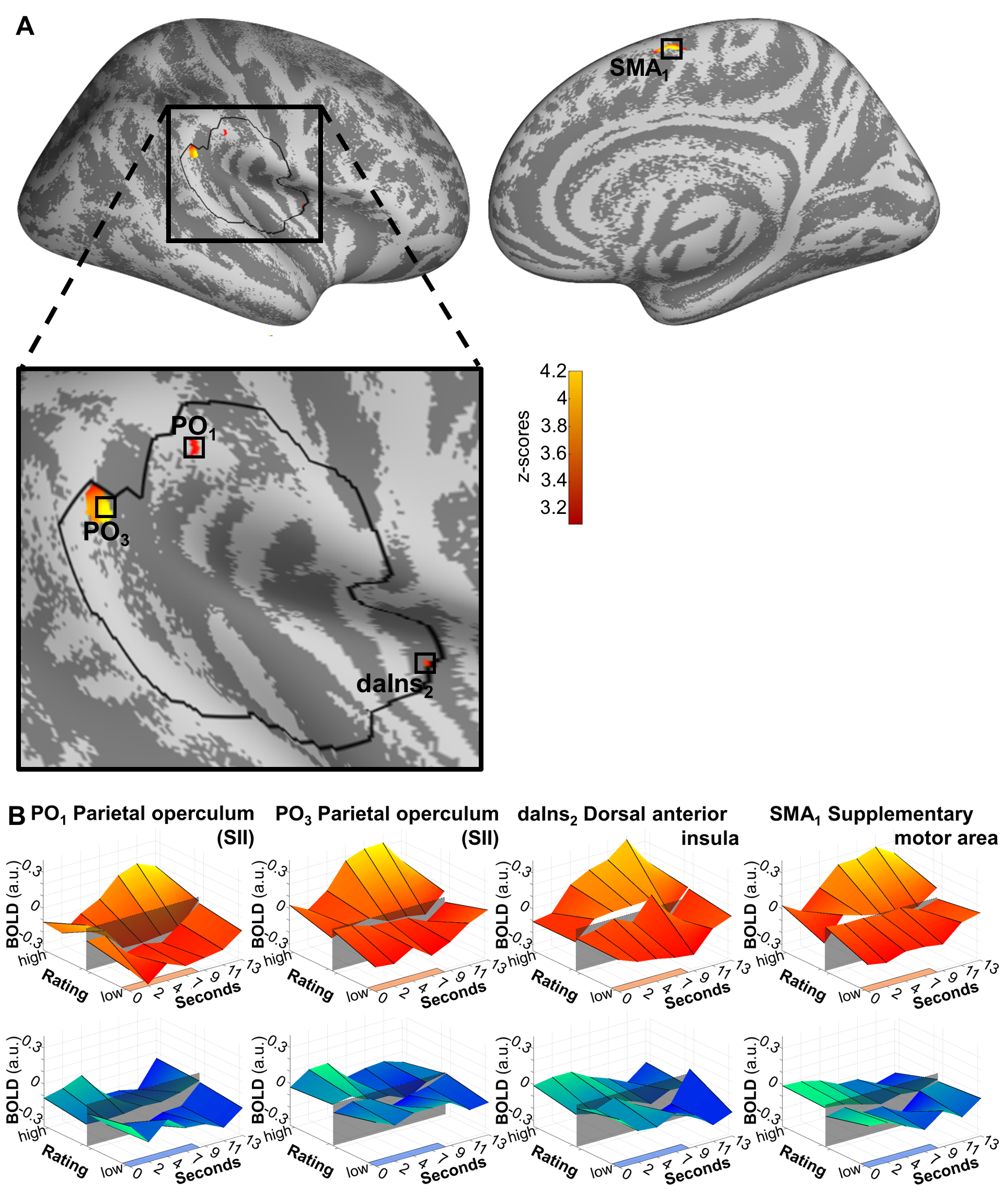

Supplement: S8 Fig — Unlike in Fig 7, activations are shown after analysis with a lower threshold of p(uncorrected) < 0.001. At this threshold, activation was found in the parietal operculum (PO3 is the cluster reported in Fig 7; PO1 is a more dorsal cluster), daIns2, and the (pre)supplementary motor area (SMA1). Exact coordinates and activation of cluster peaks were x = 55, y = −37, z = 24, Z = 4.176, p(uncorrected) = 1 × 10^−5^ (PO3), x = 61, y = −26, z = 30, Z = 3.26, p(uncorrected) = 6 x 10^−4^ (PO1), x = 35, y = 1, z = 13, Z = 3.297, p(uncorrected) = 5 × 10^−4^ (daIns2), and x = 7, y = −5, z = 60, Z = 4.202, p(uncorrected) = 1 × 10^−5^ (SMA1). (A) Activations are thresholded at p(uncorrected) < 0.001 and overlaid on an average brain surface. The black line delineates the SVC mask. (B) Poststimulus plots of fMRI activation in the 4 vertices during heat (orange) and sound (blue). The shaded patch in the center signifies the pain threshold (for heat) and unpleasantness threshold (for sound). The colored patches at the right axes show the stimulus duration. Data used to produce the figure can be found at https://www.doi.org/10.17605/OSF.IO/QXCNW. daIns2, dorsal anterior insula cluster 2; fMRI, functional magnetic resonance imaging; PO1, parietal operculum cluster 1; PO3, parietal operculum cluster 3; SMA1, supplementary motor area cluster 1; SVC, small-volume correction. (TIF) [file pbio.3000205.s011.tif]

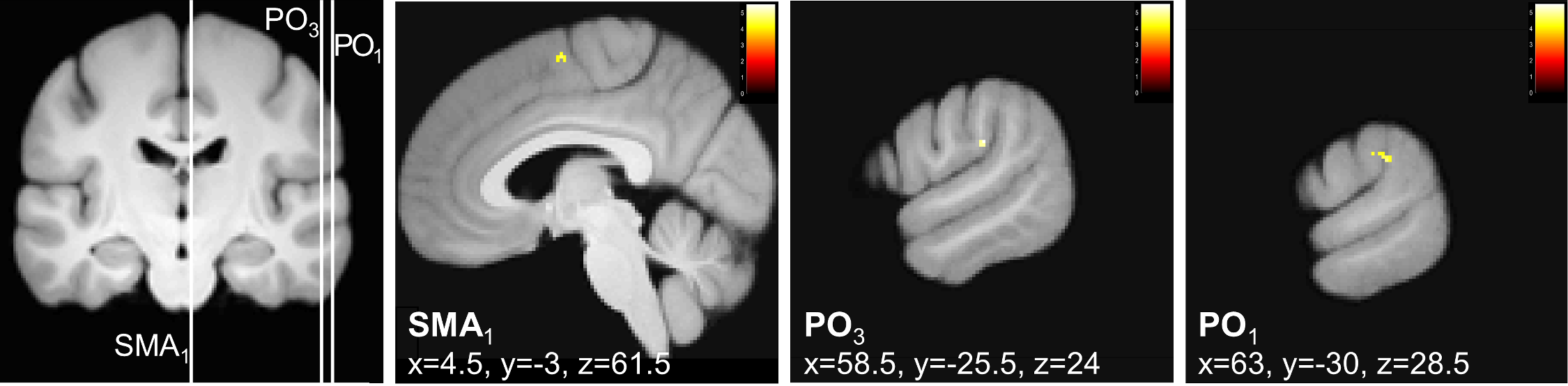

Supplement: S9 Fig — Also see Fig 7 and S8 Fig. No activations were significant at our predefined threshold (p < 0.05, corrected). For reasons of completeness, this analysis is reported at a lower threshold of p(uncorrected) < 0.001. The location of activations roughly correspond to the surface analysis presented in S8 Fig. Activation was observed in the parietal operculum (PO3 is the cluster reported in Fig 7; PO1 is a more dorsal cluster) and the (pre)supplementary motor area (SMA1), among other areas. Exact coordinates and activation of cluster peaks were x = 59, y = −26, z = 24, Z = 3.9, p(uncorrected) = 5 × 10^−5^ (PO3), x = 63, y = −30, z = 29, Z = 3.487, p(uncorrected) = 2 × 10^−4^ (PO1), and x = 5, y = 3, z = 62, Z = 3.297, p(uncorrected) = 3 × 10^−4^ (SMA1). Activations are thresholded at p(uncorrected) < 0.001 and overlaid on an average brain. PO1, parietal operculum cluster 1; PO3, parietal operculum cluster 3; SMA1, supplementary motor area 1. (TIF) [file pbio.3000205.s012.tif]

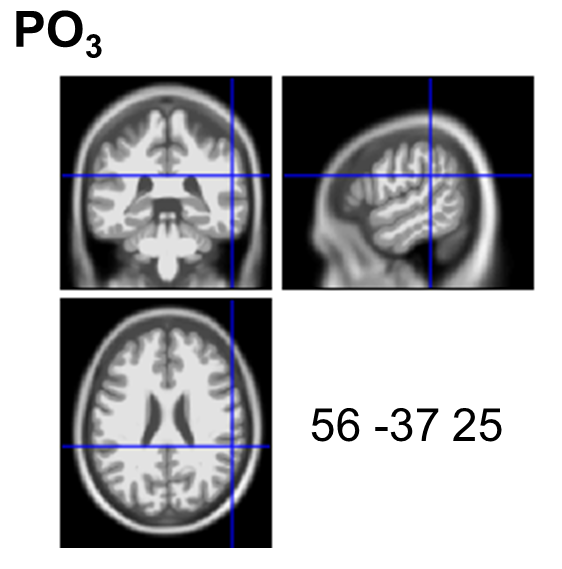

Supplement: S10 Fig — Also see Fig 7. PO3, parietal operculum cluster 3. (TIF) [file pbio.3000205.s013.tif]
